# Caspase-2 Is Upregulated after Sciatic Nerve Transection and Its Inhibition Protects Dorsal Root Ganglion Neurons from Apoptosis after Serum Withdrawal

**DOI:** 10.1371/journal.pone.0057861

**Published:** 2013-02-25

**Authors:** Vasanthy Vigneswara, Martin Berry, Ann Logan, Zubair Ahmed

**Affiliations:** 1 Neurotrauma and Neurodegeneration Section, School of Clinical and Experimental Medicine, University of Birmingham, Birmingham, United Kingdom; 2 Neuregenix Ltd, The Science Park, Edgbaston, Birmingham, United Kingdom; Universidade Federal do ABC, Brazil

## Abstract

Sciatic nerve (SN) transection-induced apoptosis of dorsal root ganglion neurons (DRGN) is one factor determining the efficacy of peripheral axonal regeneration and the return of sensation. Here, we tested the hypothesis that caspase-2 (CASP2) orchestrates apoptosis of axotomised DRGN both in vivo and in vitro by disrupting the local neurotrophic supply to DRGN. We observed significantly elevated levels of cleaved CASP2 (C-CASP2), compared to cleaved caspase-3 (C-CASP3), within TUNEL+DRGN and DRG glia (satellite and Schwann cells) after SN transection. A serum withdrawal cell culture model, which induced 40% apoptotic death in DRGN and 60% in glia, was used to model DRGN loss after neurotrophic factor withdrawal. Elevated C-CASP2 and TUNEL were observed in both DRGN and DRG glia, with C-CASP2 localisation shifting from the cytosol to the nucleus, a required step for induction of direct CASP2-mediated apoptosis. Furthermore, siRNA-mediated downregulation of CASP2 protected 50% of DRGN from apoptosis after serum withdrawal, while downregulation of CASP3 had no effect on DRGN or DRG glia survival. We conclude that CASP2 orchestrates the death of SN-axotomised DRGN directly and also indirectly through loss of DRG glia and their local neurotrophic factor support. Accordingly, inhibiting CASP2 expression is a potential therapy for improving both the SN regeneration response and peripheral sensory recovery.

## Introduction

The axons of dorsal root ganglion neurons (DRGN) transmit sensory information from the periphery to the CNS. Although peripheral DRGN axons regenerate, functional recovery is poor, either because axotomy induces apoptosis in DRGN or regenerating axons are misrouted and re-innervate inappropriate targets. Distinct functional as well as structural changes are observed in DRG after PNS injury [1], including initial proliferation and activation of satellite cells surrounding DRGN [2], up-regulation of neurotrophic factors and neurosteroids, alteration in the expression of neurotransmitters and apoptotic cell death of a sub-population of DRGN and their satellite cells [3]. The extent and severity of peripheral nerve injury determines the magnitude of axotomised DRGN cell death. For example, after crushing the SN most L4/L5 DRGN survive and regenerate their axons [2] whereas, approximately 15–30% of DRGN die 30 days after complete SN transection [2]. The survival of mature DRGN is moderated by hyper-glycaemia, oxidative stress [4], intracellular calcium levels [5], ionizing radiation [6] and neurosteroids [7] although the molecular mechanisms underlying axotomy-induced apoptosis remains unclear.

After peripheral axotomy, apoptosis of both DRGN and Schwann cells in DRG may be mediated by CASP3 [8], [9]. Although a role for CASP2 in DRGN apoptosis has not been identified, CASP2 does mediate apoptosis in hippocampal neurons, sympathetic neurons [10], [11], retinal ganglion cells [12], pancreatic acinar cells and insulin secreting (HIT-T15) cells [13], cancer cells [14] and murine macrophages [15]. CASP2 triggers cell death through both intrinsic mitochondrial and extrinsic death-receptor mediated pathways in response to death stimuli such as heat shock [16], DNA damage [17], generation of reactive oxygen species (ROS) [11], cytotoxic and endoplasmic reticulum (ER) stress [18] and cytoskeletal disruption [19].

In the current study, we aimed to determine if CASP2 mediates axotomy-induced apoptosis of SN transected adult DRGN *in vivo* and in the well-established serum withdrawal *in vitro* model of DRGN axotomy.

## Materials and Methods

### Ethics statement

This study was carried out in strict accordance to the UK Animals Scientific Procedures Act, 1986 and all procedures were licensed and approved by the UK Home Office. The protocols and experiments were also approved by the University of Birmingham Ethical Review Sub-Committee. All surgery was performed under inhalation anaesthesia using 5% Isofluorane (IsoFlo, Abbott Animal Health, North Chicago, IL, USA) for induction and 2% for maintenance. Animals were kept in environmentally controlled animal facilities at the University of Birmingham and every effort was made to minimise animal suffering.

### In vivo DRGN injury models

To investigate the role of CASP2 in DRGN apoptosis, SN injury models were established as described previously [20]. SN were transected unilaterally in adult female Sprague-Dawley rats (150–200 g) at the level of the sacrotuberous ligament. Spontaneous regeneration of SN was inhibited by excising a 3 mm segment of distal nerve stump, ligating both nerve stumps and burying the proximal stump deep into gluteus maximus muscle. Animals were allowed to survive for 1 month, intracardially perfused with 4% formaldehyde (TAAB Laboratories, Peterborough, UK), the L4/L5 DRG harvested, cryoprotected and blocked up in OCT mounting compound (TAAB Laboratories) and stored at −20°C until required. For all experiments, we used L5 DRG sections for immunohistochemistry and analysis.

### Immunohistochemistry

Longitudinal 15 µm thick sections of L5 DRG were cut using a Bright cryostat (Bright Instruments, Huntingdon, UK), and adhered onto charged glass slides and immunohistochemistry performed as described by us previously [12], [21]. Briefly, sections were fixed in 100% ethanol, washed ×3 in phosphate buffered saline (PBS), permeabilised in PBS containing 1% Triton X-100 (Sigma, Poole, UK) and blocked in PBS containing 0.5% bovine serum albumin (BSA) (Sigma) and 0.05% Tween 20 (PBS-T-BSA). Sections were then incubated overnight (16–18 h) at 4°C in a humidified chamber with the relevant primary antibody diluted appropriately in PBS-T-BSA. CASP2 and C-CASP2 was detected with a with polyclonal anti-rabbit antibody (Abcam, Cambridge, UK) while C-CASP3 was detected with a rabbit polyclonal antibody (Cell Signalling Technology, Danvers, MA, USA) while Schwann cells were labelled with p75^NTR^ (Sigma), all diluted at 1∶200 in PBS containing BSA. Sections were then washed ×3 in PBS and incubated with the relevant secondary antibody diluted in PBS-T-BSA for 1 h at room temperature. For fluorescent detection of the antigen, secondary antibodies were either coupled to Alexa Fluor 488 (Green) or Texas Red (Red) (both from Molecular Probes, Oregon, USA), and diluted at 1∶400. Coverslips were mounted in Vectamount with and without DAPI (Vector Labs, Peterborough, UK) and viewed under a fluorescent microscope (Carl Zeiss, Welwyn-Garden City, UK). Controls were incubated in each run for each antibody tested, including omission of the primary antibody and, where available, blocking peptides were pre-incubated with the primary antibody at a ratio of 1∶20 for 2 h before incubation. Control sections showed no positive immunohistochemistry for relevant antibodies. As a minimum, each antibody run was performed in duplicate and performed on 3 independent occasions using tissue sections from 6 different animals.

Images from immunohistochemistry were captured using Axiovision Software and an AxioCam HRc attached to the fluorescent microscope (all from Zeiss). Negative controls were used to set the threshold level to account for background fluorescence levels (noise) and therefore not shown.

### Preparation of adult DRG cultures

Primary DRG cultures were prepared using 6–8 week-old female Sprague-Dawley rats (150–200 g) (Charles River, Margate, UK) killed by CO_2_ exposure, as described by us previously [21], [22], [23]. Briefly, L4–L7 DRG pairs were dissected, dissociated into single cells in a solution of Neurobasal-A containing 0.1% collagenase (Sigma) and 200 U/ml DNaseI (Worthington Biochem, New Jersey, USA). Dissociated DRGN were pelleted and re-suspended in Dulbecco's Modified Eagle Medium (DMEM) (Invitrogen) containing 5-Flouro-2-deoxyuridine (FDU-to limit glial proliferation) (Merck Biosciences, Feltham, UK) at a final concentration of 30 µM and 1% Penicillin-Streptomycin (P/S), with and without 10% fetal bovine serum (FBS) (Invitrogen) and plated at a cell density of 300 cells/well on sterile glass chamber slides (BD Biosciences, Oxford, UK) pre-coated with 100 µg/ml poly-D-lysine followed by 20 µg/ml Laminin-I (both from Sigma) and cultured for 72 h at 37°C in a humidified atmosphere containing 5% CO_2_.

### Immunocytochemistry

To detect DRGN survival and expression of CASP2, CASP3, C-CASP2 and C-CASP3, cultures were fixed in 4% formaldehyde for 10 min, washed ×3 in PBS, blocked in PBS-T-BSA (3% BSA and 0.1% Triton X-100) (Invitrogen) for 10 min at room temperature and incubated with polyclonal anti-rabbit cleaved CASP2 (Abcam) or rabbit polyclonal C-CASP3 (Cell Signalling Technology) and monoclonal anti-mouse βIII-tubulin (Sigma), all diluted at 1∶200 in PBS containing BSA. Cells were then washed ×3 in PBS and incubated with anti-mouse Alexa Fluor 488 (Green) and anti-rabbit Alex Flour 594 (Red) (Invitrogen) diluted 1∶400 in PBS-BSA for 1 h at room temperature. After the final 3 washes with PBS, cover slips were mounted in Vectamount with or without DAPI (Vector Laboratories) and viewed under a fluorescent microscope (Zeiss). To assess antibody specificities of caspases, negative controls were included in each run for each antibody tested including omission of the primary antibody.

A blinded observer divided each 8-well chamber into 9 quadrants and randomly took photomicrographs from each quadrant using a fluorescent microscope (Zeiss) attached to a computer running the Axiovision software (Zeiss). The observer then counted the number of βIII-tubulin^+^ DRGN with DAPI stained nuclei to assess the degree of survival in each relevant experiment (n = 54 fields/condition). Experiments were performed in duplicate and repeated on at least 3 independent occasions.

### siRNA-mediated knockdown of CASP2 and CASP3

Dissociated DRGN were allowed to resettle for 20–24 h in DMEM containing 30 µM FDU (Merck) with 10% FBS at 37°C in a humidified atmosphere containing 5% CO_2_ prior to transfection using Lipofectamine 2000 reagent (Invitrogen) according to the manufacturer's instructions for knocking down mRNA for both CASP2 and CASP3 using 10 nM of a chemically modified siRNA (siCASP2; Gift from Quark Pharmaceuticals, Israel) and scrambled (Scr-siCASP2) to CASP2 mRNA [12] and chemically synthesised siRNA (siCASP3) (anti-sense: 5′–AATGGTACCGATGTCGATGCA–3′; sense oligonucleotide: 5′–AATGCATCGACATCGGTACCA–3′ and scrambled (Scr-siCASP3; anti-sense: AACTACGTGAGTGAGTCTGAC; sense: AAGTCAGACTCACTCACGTAG) targeting CASP3 mRNA (synthesized by Alta Bioscience, University of Birmingham, UK).

Controls included DRG cultures transfected with Lipofectamine alone and a non-specific siRNA to green fluorescent protein (siGFP) (Dharmacon, Lafayette, CO, USA) and scrambled versions of siCASP2 and siCASP3. After transfection for 5 h, serum free DMEM containing 30 µM FDU to limit glial cell proliferation and acting as a potential source of neurotrophic factors [23] was added to the transfection medium to make up the final volume up to 500 µl/chamber. Finally DRG cultures were incubated for a further 72 h at 37°C in a humidified atmosphere containing 5% CO_2_, before either fixation in 4% formaldehyde and subsequent immunocytochemistry (described above) or cell lysis for western blot analysis as described in the next section. Each knockdown experiment was performed in duplicate and repeated on at least 3 independent occasions. Levels of knockdown were quantified by densitometry as described in the next section.

### Protein extraction, western blotting and densitometry

To determine the levels of CASP2 and -3 protein after siRNA-mediated knockdown, DRG cultures were washed ×2 in PBS and incubated in ice-cold lysis buffer containing 20 mM Tris-HCL (pH 7.4), 1 mM EDTA, 0.5 mM EGTA, 150 mM NaCl, 1% NP-40 (Sigma) and protease inhibitor cocktail (Sigma), incubated on ice for 30 min, and centrifuged at 13,000 rpm at 4°C, as described by us previously [23]. Lysates were normalised for protein using a calorimetric DC assay kit (Bio-Rad, Hercules, CA, USA) and 10 µg of total protein were resolved on 12% SDS-polyacrylamide gels. Proteins were transferred to PVDF membranes (Millipore, Gloucester, UK), blocked for 1 hr and blotted overnight using the relevant antibody. Resolved bands were detected using a enhanced chemiluminescence (ECL) system (GE Healthcare, Buckinghamshire, UK) and relevant HRP-conjugated secondary antibodies (1∶1000; GE Healthcare). Each blot was stripped and re-probed with β-actin antibody as a protein loading control. Each knockdown experiment was repeated on 3 independent occasions.

To quantify detected bands by densitometry, blots were scanned into Photoshop (Adobe Systems, San Jose, CA, USA) and analysed using the built-in gel plotting macros in ScionImage (Scion Corporation, Maryland, USA) [23].

### TUNEL assay and counter staining

Apoptotic DNA fragmentation was detected in tissue cryosections and DRG cultures using a fluorescent TUNEL assay kit (Merck, Feltham, UK and Millipore, Billerica, MA, USA) according to the manufacturer's instructions. Briefly, after permeabilization with Proteinase-K, cryosections were incubated with TUNEL reaction mixture containing terminal deoxynucleotidyl transferase (TdT) and dUTP coupled to fluorescein for 1 h at 37°C. Positive controls were generated using DNAase I digestion, as described by the manufacturer. Counterstaining for C-CASP2 was performed as described under the “Immunohistochemistry” section with some modification in the protocol followed by the TUNEL staining. However, for the double immunofluorescent and TUNEL staining of DRG culture, cells were fixed with 4% formaldehyde, permeabilized with 0.1% Triton X-100 and the rest of the procedure was performed similarly to that described above for immunohistochemistry. Each TUNEL assay was performed in triplicate using sections from at least 6 different animals in each run. TUNEL assay in cell culture was performed in duplicate and repeated on 3 independent occasions.

### Quantification of the number of TUNEL^+^ DRG neurons, satellite and Schwann cells

Three sections from the mid-point of each DRG (n = 3 DRG/treatment), determined from the total number of 15 µm-thick sections, were stained with the TUNEL kit and used to count the number of TUNEL^+^ DRGN, satellite and Schwann cell nuclei after image capture using Image Pro software (Media Cybernetics, Bethesda, MD, USA) in the whole DRG section. DRG glia were defined as satellite cells if they tightly surrounded a DRGN and as Schwann cells if spindle shaped and within the DRG neuropil un-associated with blood vessels. The total number of DAPI stained DRGN and DRG glia nuclei were also counted using Image Pro (Media Cybernetics) and the number of TUNEL^+^ DRGN and DRG glia were expressed as a percentage of the total number of cells in an entire section of DRG.

### Statistical analysis

Sample means were calculated and tested for significance using ANOVA with post-hoc testing by Dunnet's method.

## Results

### SN transection induced up-regulation of C-CASP2 and apoptosis of DRGN and their satellite cells

**Figure 1:**
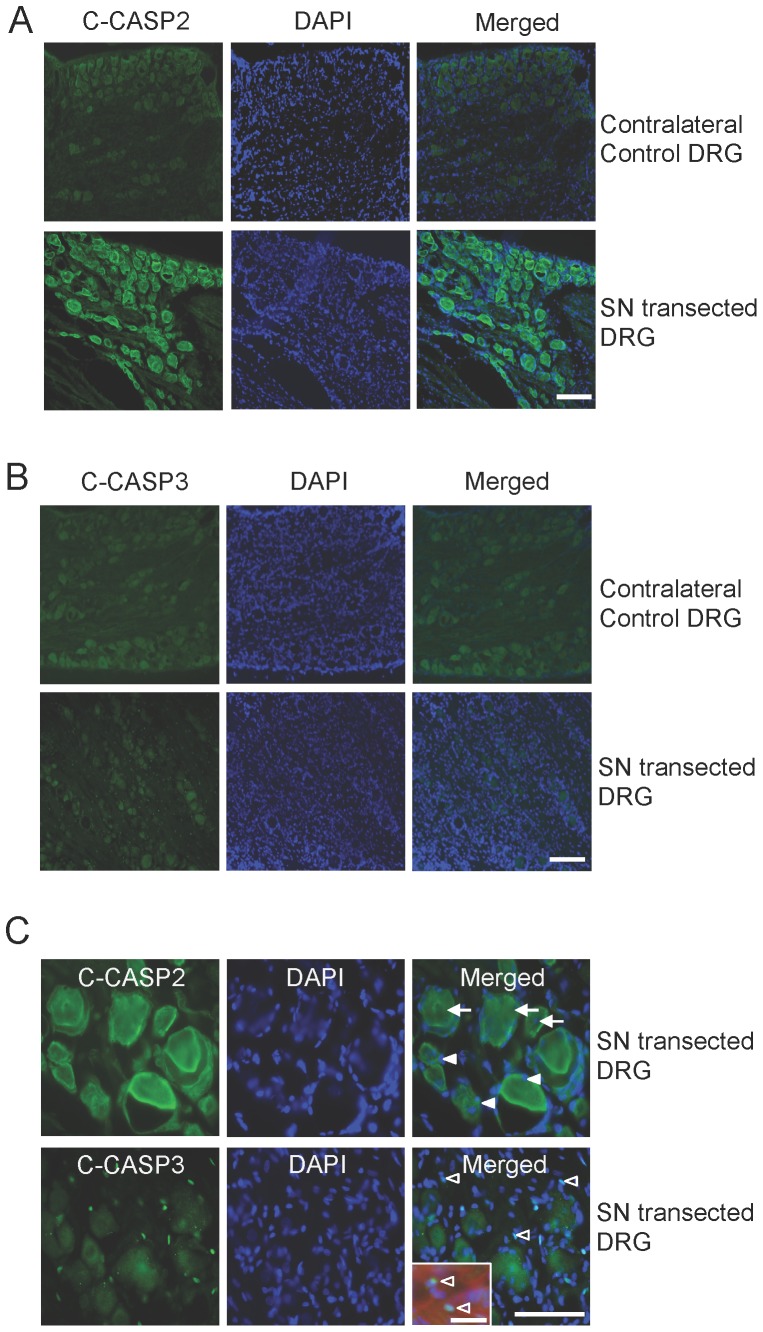
C-CASP2 is highly induced in DRGN and satellite cells after SN transection. ***A***, ***B***, Constitutive low levels of C-CASP2 and C-CASP3 in control DRGN and satellite cells, while high levels of C-CASP2 compared to C-CASP3 were observed in DRGN and satellite cells 1 month after SN transection. ***C***, High power magnification showing C-CASP2 and C-CASP3 in DRGN (arrows) and satellite cells (arrowheads) and C-CASP3 in Schwann cells (open arrowheads). Inset in (**C**) shows high power magnification of double immunohistochemistry for p75^NTR^ and C-CASP2. Scale bar in *A*–*C* = 50 µm.

**Figure 2:**
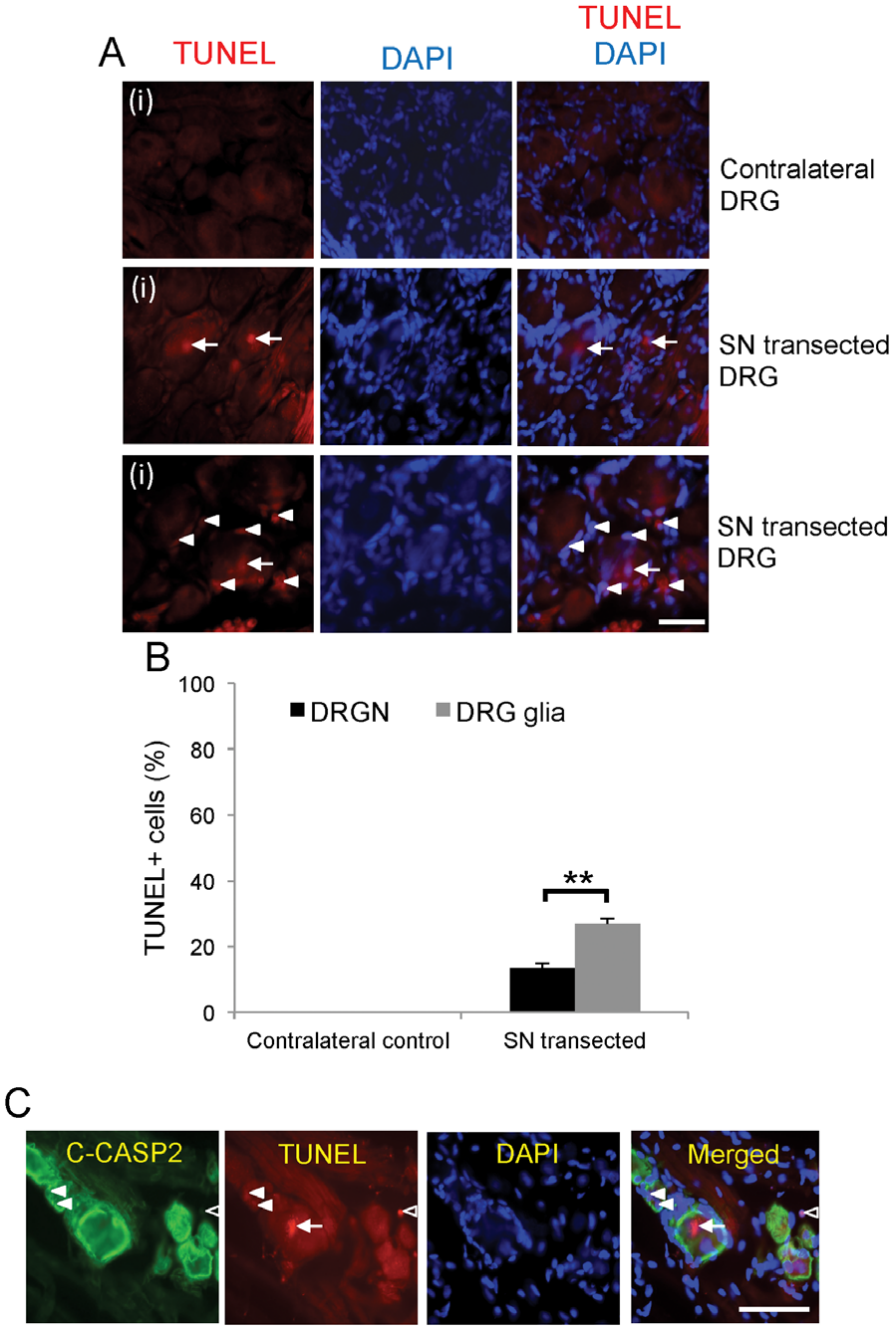
Apoptosis of DRGN and satellite cells at 1 month after SN transection. ***A***, TUNEL staining in sections from contralateral control (**i**) and axotomised DRGN (**ii**) and (**iii**) ((**ii**) shows 2 TUNEL^+^ DRGN nuclei, while (iii) shows TUNEL^+^ DRGN and DRG glia in an adjacent area)), 1 month after SN transection. DRGN (arrows) and satellite cell (arrowheads) nuclei were TUNEL^+^ while contralateral DRGN and satellite cells were TUNEL^−^. ***B***, Relative percentages of TUNEL^+^ DRGN and DRG glia (satellite+Schwann cells) in ipsilateral and contralateral DRG after SN transection. ***C***, Double labelling for TUNEL and C-CASP2 in DRGN (arrows), satellite cells (arrowheads) and Schwann cells in the DRG neuropil (open arrowheads). Scale bars in *A*–*C* = 50 µm. **p<0.001, ANOVA.

There was a constitutive low level of C-CASP2 (Figure 1A) and C-CASP3 (Figure 1B) immunolocalisation in contralateral control DRGN and satellite cells. Thirty days after SN transection, enhanced C-CASP2 (Figure 1A (ii), (iii) and C) and lower levels of C-CASP3 were localised in DRGN and satellite cells (Figure 1B). In addition, spindle-shaped cells un-associated with blood vessels and DRGN, morphologically identical to Schwann cells, were strongly C-CASP3^+^ (Figure 1C; open arrows and inset). Ten% of total DRGN (Figure 2A; arrows) and 30% of DRG glia (Figure 2A: arrowheads) were TUNEL^+^ after SN transection (Figure 2B), suggesting their co-dependence and that death of both types of cells was mediated by caspase-2, while CASP3 mediated Schwann cell death only (Figure 1C; open arrows). All TUNEL^+^ DRGN (arrow) and satellite cell (arrowheads) nuclei were also C-CASP2^+^ (Figure 2C).

### Serum withdrawal activates caspase-2 and is associated with apoptotic death of DRGN and DRG glia

**Figure 3:**
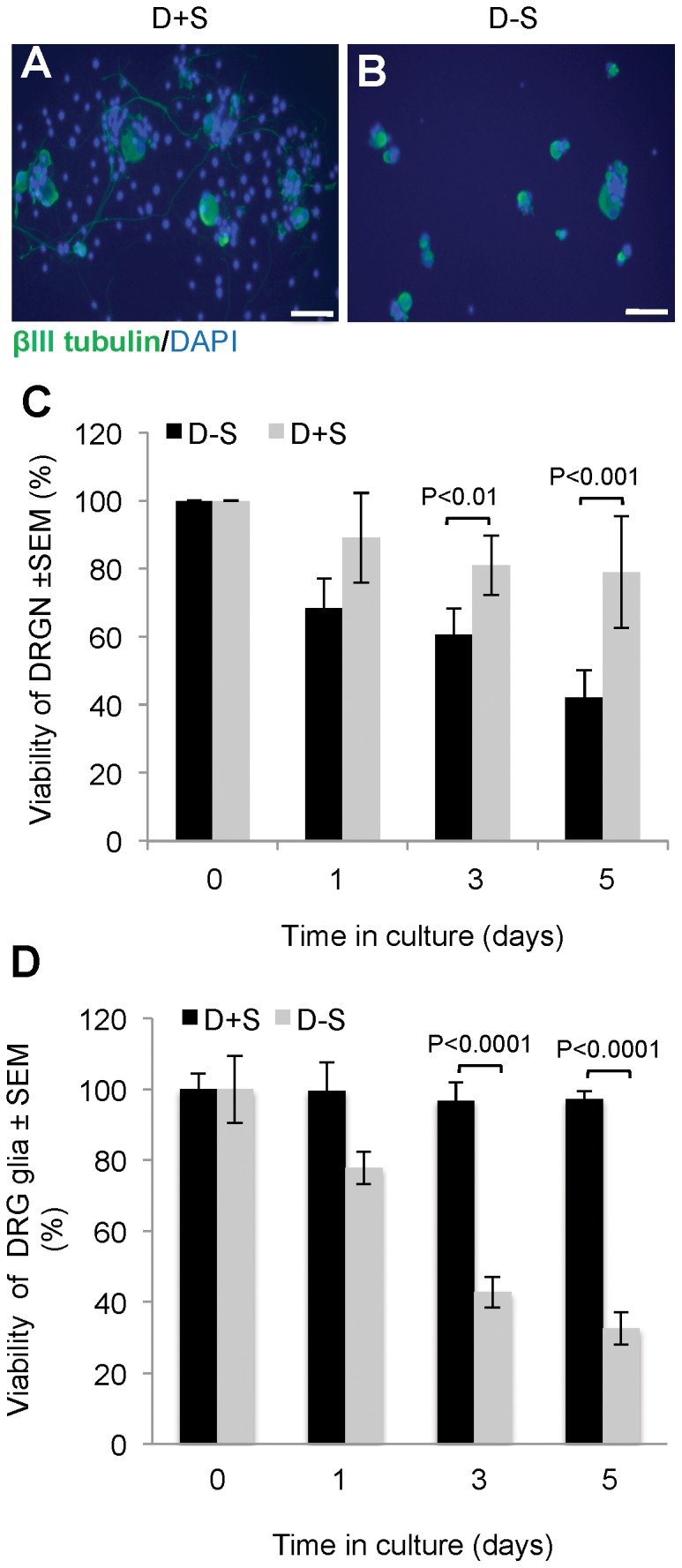
Survival of DRGN after 5 days in culture, in the presence of serum and after serum withdrawal. DRGN grown in ***A***, presence of serum (D+S) or, ***B***, after serum withdrawal (D−S). ***C***, DRGN and ***D***, DRG glia survival in the presence of serum and after serum withdrawal, over time in culture. The data presented here are means±SEM from 3–5 separate experiments conducted in duplicate.

To test the possibility that CASP2 is important in mediating the death of DRGN and DRG glia, we used the serum-withdrawal DRG cell culture model. DRGN cultured in medium containing serum (D+S) had long neurites (Figure 3A), while those grown in serum free medium (D−S) failed to grow neurites (Figure 3B) and eventually died (Figure 3C). After 3 days in culture, 40% of the total number of plated DRGN had died, rising to nearly 60% by 5 days (Figure 3C). DRG glia were more susceptible to death induced by serum withdrawal and, by 3 days in culture, >60% of the total number of cells had died (Figure 3D). These results demonstrated that serum withdrawal caused a time-dependent decrease in DRGN survival and neurite outgrowth together with death of DRG glia. Hence, exogenous trophic support is required for DRGN survival and neurite outgrowth and also for DRG glia survival.

### Localisation of CASP-2, -3, C-CASP-2 and -3 and TUNEL in both DRGN and satellite cells

**Figure 4:**
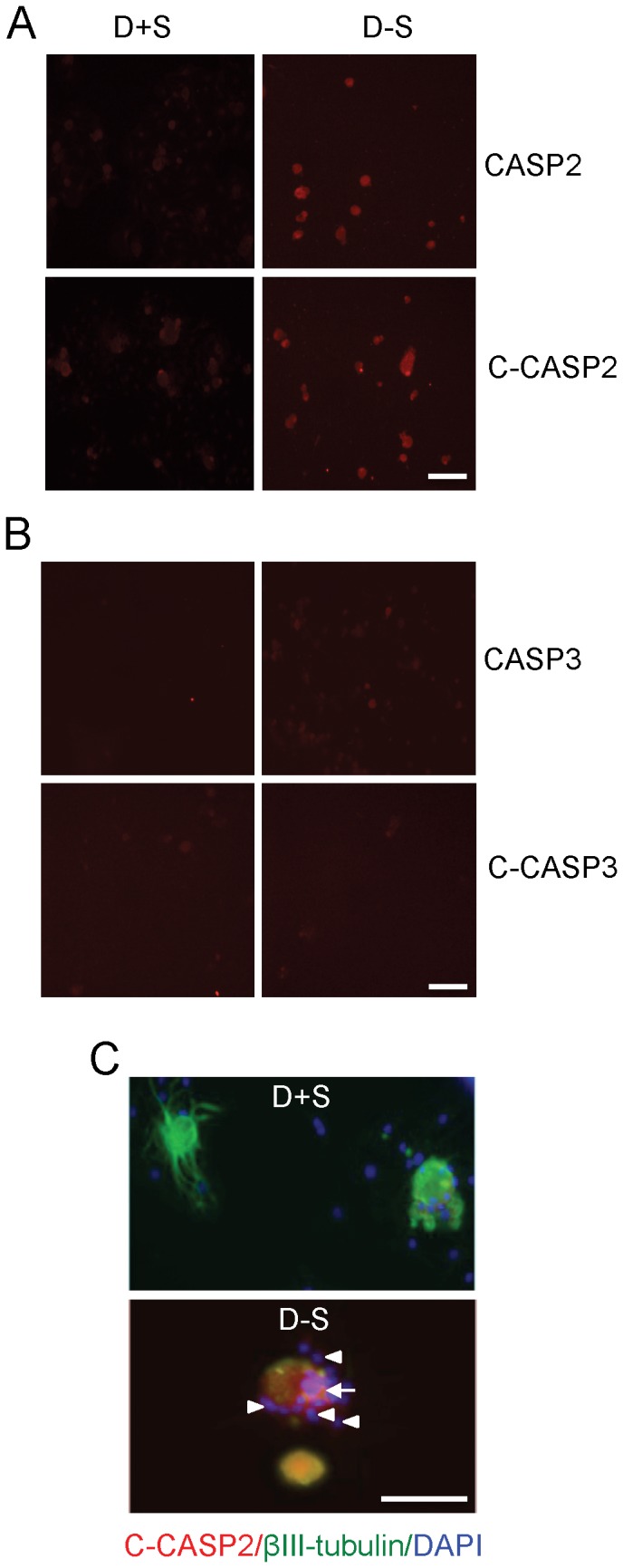
CASP2 is activated in serum-withdrawn DRGN and satellite cells. ***A***, CASP2, C-CASP2 and ***B***, CASP3, C-CASP3 in DRGN and satellite cells grown in the presence of serum (D+S) or after serum withdrawal (D−S). ***C***, Double immunostaining for C-CASP2 in βIII-tubulin^+^ DRGN (arrow) and C-CASP2^+^ in βIII-tubulin^−^ satellite cells (arrowheads). Scale bars in *A*–*C* = 50 µm.

**Figure 5:**
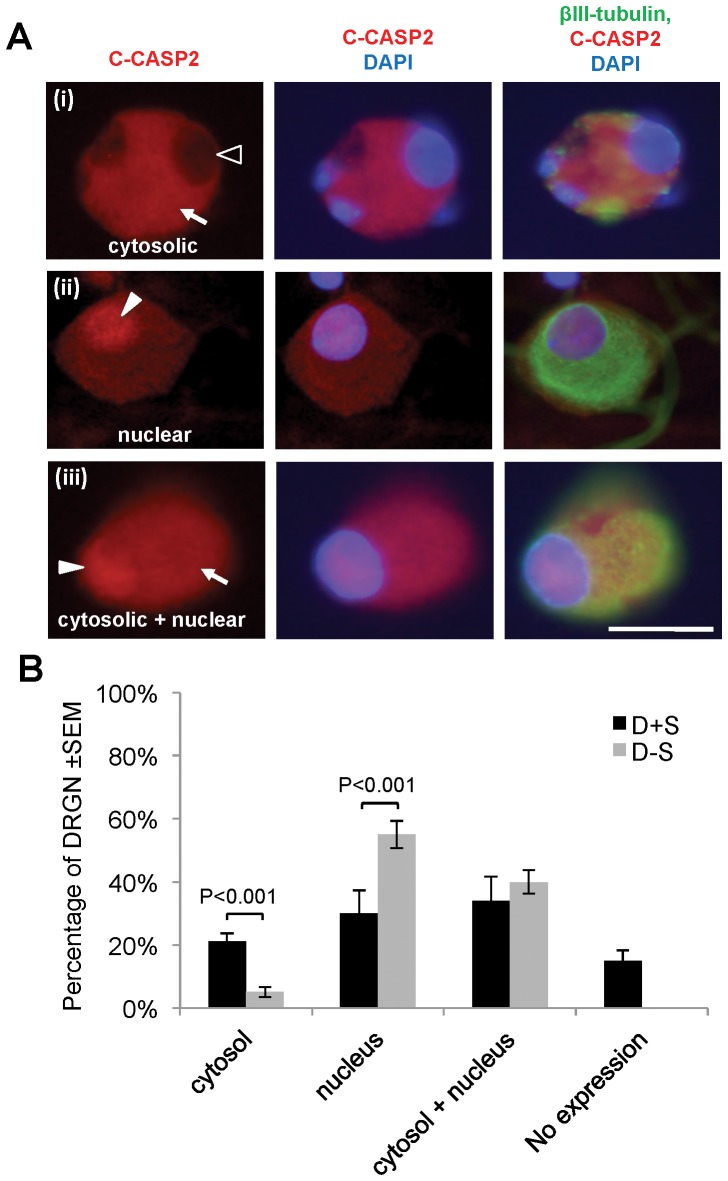
Localisation of C-CASP2 to the cytosol, nucleus, and to both compartments either in the presence of serum or after serum withdrawal. The patterns of C-CASP2 immunocytochemistry included cytosolic (arrow) but not nuclear expression (open arrowhead) *A(i)*, nuclear (closed arrowhead) *A(ii)*, or cytosolic (arrow) and nuclear (arrowhead) *A(iii)*. ***B***, Percentage frequency of DRGN with either cytosolic, nuclear or cytosolic+nuclear localisation of C-CASP2 in the presence of serum (D+S) and after serum withdrawal (D−S). Scale bar = 50 µm.

**Figure 6:**
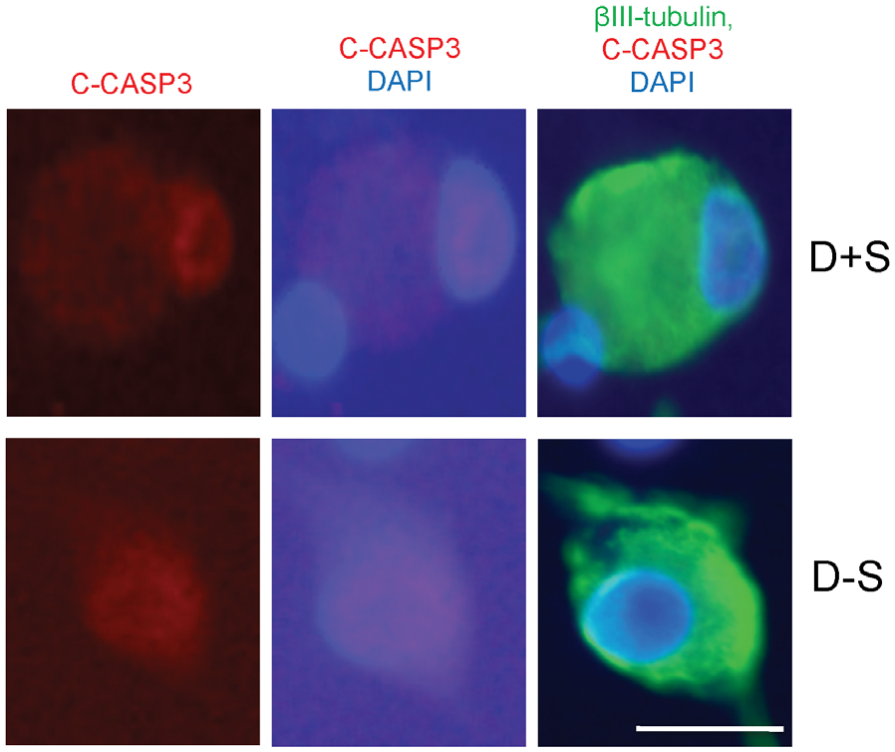
High power images demonstrating immunostaining for C-CASP3 and its localization in cytosol and nucleus. C-CASP3 localisation in DRGN grown in both the presence of serum (D+S) and after serum withdrawal (D−S). Scale bar = 50 µm.

**Figure 7:**
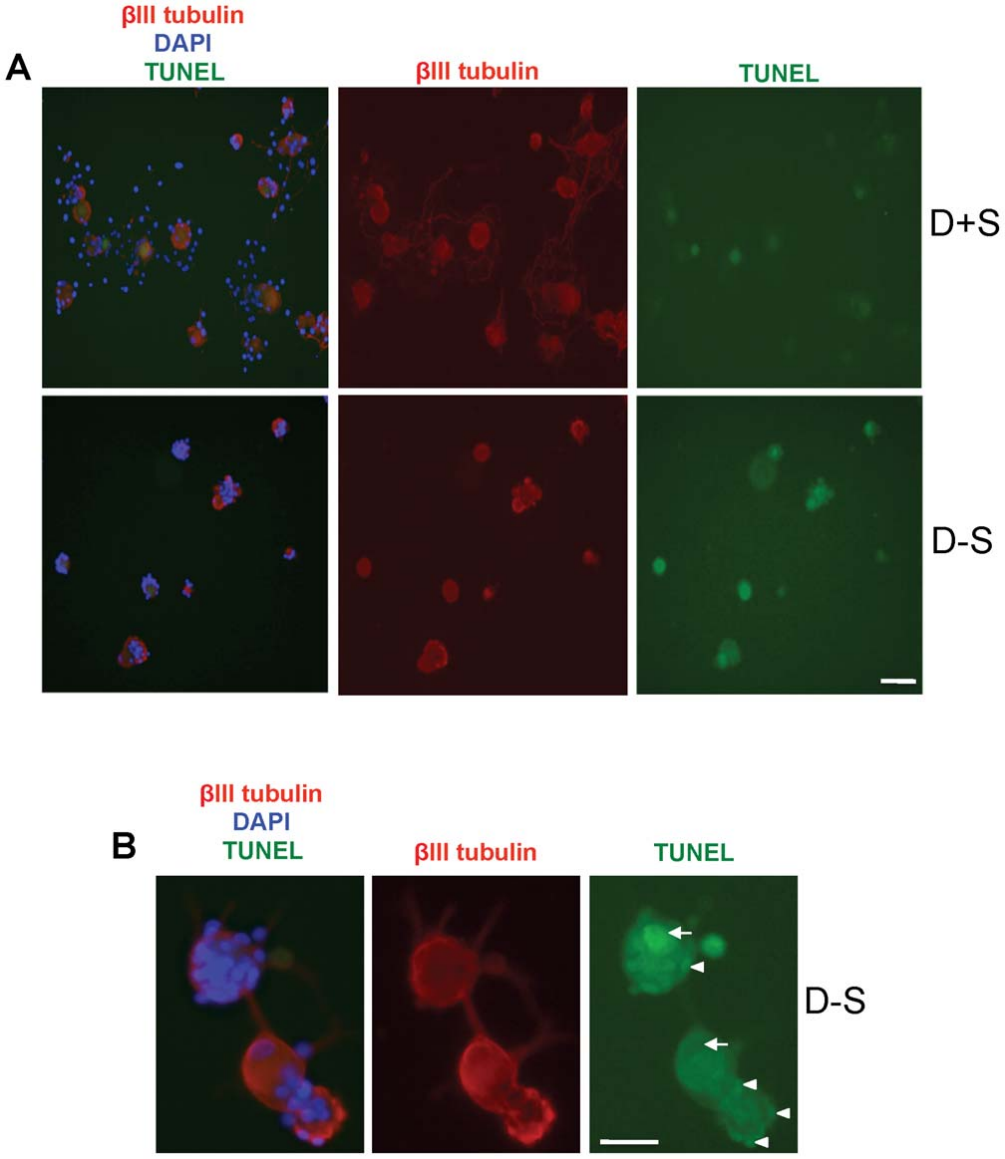
TUNEL staining in 3 day DRG cultures grown in DMEM in the presence of serum (D+S) and after serum withdrawal (D−S). ***A***, TUNEL^+^ DRGN in the presence of serum (D+S) or after serum withdrawal (D−S). ***B***, DRGN grown in the absence of serum (D−S) demonstrating TUNEL^+^ nuclei of both DRGN (arrow) and satellite cells (arrowheads). Scale bars in *A* and *B* = 50 µm.

Serum withdrawal increased CASP2 and C-CASP2 levels in DRGN (Figure 4A), while little or no change in either CASP3 or C-CASP3 (Figure 4B) was detected in serum starved compared to serum nurtured DRGN. There was little or no C-CASP2^+^ staining in either DRGN or DRG glia grown in the presence of serum (D+S) (Figure 4C). However, after serum withdrawal (D−S), C-CASP2 levels rose in DRGN (arrow) and their associated satellite cells (arrowheads) (Figure 4C). C-CASP2 and C-CASP3 in DRGN were localised to within either the cytosol (Figure 5A(i)), nucleus (Figure 5A(ii)) or in both compartments (Figure 5A(iii)). DRGN numbers fell after serum withdrawal and correlating with a concomitant 50% increase in nuclear C-CASP2 localisation (Figure 5B). Immunostaining for C-CASP3 on the other hand, was weak in DRGN cytosol and nucleus and no obvious differences in the intracellular localisation were observed between serum-withdrawn (D−S) and serum fed DRGN (D+S) (Figure 6). These data in an *in vitro* paradigm of exogenous neurotrophic factor deprivation in DRGN, suggest that serum withdrawal causes activation of CASP2 and not CASP3 in DRGN and satellite cells. Greater numbers of DRGN and their satellite cells were TUNEL^+^ after serum withdrawal (D−S) than those grown in the presence of serum (D+S) (Figure 7A and 7B).

### siRNA meditated knockdown of CASP2, but not CASP3, rescued DRGN and DRG glia from apoptosis

**Figure 8:**
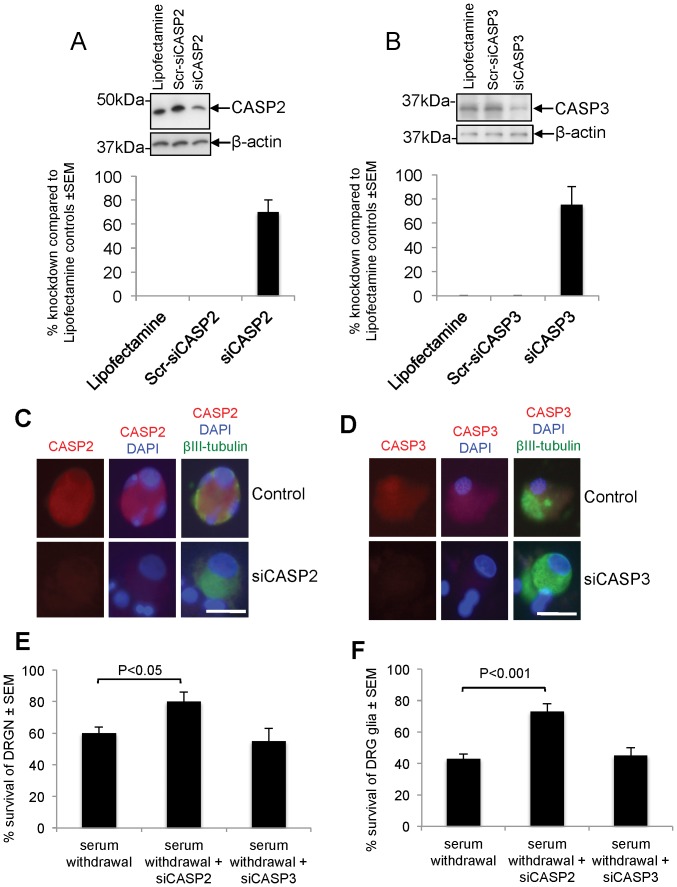
CASP2 knockdown protects DRGN and DRG glia from apoptosis induced by serum withdrawal. ***A***, Western blot and ***B***, subsequent densitometry to show CASP2 and CASP3 protein levels after knockdown with relevant siRNA. ***C***, and ***D***, Immunocytochemistry to demonstrate CASP2 and CASP3 knockdown in DRGN and satellite cells by relevant siRNA, respectively. ***E***, Quantification of DRGN and ***F***, DRG glia survival after siRNA-mediated knockdown of CASP2 and CASP3. Scale bars in *C* and *D* = 50 µm.

Since elevated levels of CASP2 were seen in cultured adult DRGN and DRG glia after serum withdrawal, we used siRNA-mediated gene silencing of CASP2 and CASP3 to determine which of these protected DRGN from death after serum withdrawal. Approximately 65% and 75% gene silencing was achieved in DRG cultures after siCASP2 and siCASP3 treatment, respectively (Figure 8A and 8B). Lipofectamine, Scr-siCASP2 and Scr-siCASP3 control transfections did not affect either CASP2 or CASP3 protein levels, demonstrating the specificity of the siRNA sequences (Figure 8A and 8B). Knockdown of the relevant protein in DRG cultures also reduced immunohistochemical levels of CASP2 and -3 in DRGN and DRG glia after relevant siRNA treatments (Figure 8C and 8D). Knockdown of CASP2 mRNA by RNAi protected 50% of both DRGN (Figure 8E) and DRG glia (Figure 8F) from apoptosis induced by serum withdrawal, while knockdown of CASP3 had no effect. These results suggest that CASP2 and not CASP3 orchestrates DRGN and DRG glia apoptosis in *in vitro* after serum withdrawal.

## Discussion

A greater frequency of immature DRGN undergo axotomy- and NGF-induced apoptotic cell death compared to adult DRGN [24]. However, it is noteworthy that, despite the ability of sensory neurons to regenerate axons, experimentally induced chronic sciatic nerve injuries cause significant levels of death of adult DRGN and incomplete functional recovery [2], [20], [25]. Approximately 40% of DRGN die within 2 months in animal models of peripheral axotomy and a similar loss is also observed clinically; indicating that the death of sensory neurons is one of the key determinants for the poor sensory recovery after SN injury [2], [20], [25]. Although the molecular mechanisms underlying axotomy-induced DRGN death are not fully understood, the extent and severity of the axonal injuries and the loss of available target-derived exogenous trophic factors are believed to significantly impact on apoptosis and the potential for axon regrowth [25], [26]. Despite the fact that mature sensory neurons are less dependent than immature neurons upon trophic support for their survival, neurotrophins still regulate survival and axon regeneration after injury [27]. In clinical situations where target re-innervation is delayed or absent (e.g. brachial plexus lesions), substantial DRGN apoptosis is a likely scenario [28], [29]. Our model of SN transection and blockade of invasion of the distal nerve stump mimics this and induces similar DRGN loss. In this study, we investigated the role of CASP2 in axotomy-induced DRGN apoptosis, by limiting the exogenous availability of target-derived neurotrophic factors.

Consistent with the study of Hart and co-workers (2002) [29], we observed a similar magnitude of DRGN death (10%), 1 month after SN transection, accompanied by 30% loss of DRG glia. In our SN transection model, the combined TUNEL- and immunostaining with antibodies against C-CASP2 and C-CASP3 revealed co-activation of CASP2 only, correlating with DRGN, satellite cell and Schwann cell apoptosis. Levels of C-CASP2 were much higher than C-CASP3 in DRGN and DRG glia, suggesting that CASP2 may have the more significant role in the apoptotic cascade. Despite the fact that many different DRGN reside in the DRG, characterised by parameters such as neuronal size, target profile and whether myelinated or unmeyelinated [30], [31], almost all DRGN showed raised C-CASP2 and were C-CASP2^+^. This suggests that all sizes of DRGN were similarly affected by trophic factor withdrawal. In addition, the 30% increase in TUNEL^+^ satellite cells around dying DRGN indicated an inter-dependence of satellite cells with DRGN, linking satellite cell survival with axotomised DRGN by restricting the supply of local neurotrophic factors [2], [7].

The observed survival inter-dependence of DRGN and satellite cells suggest that signals retrogradely transported in DRGN axons are a regulatory stimulus for the satellite cell integrity underlying DRGN-satellite cell interactions. Satellite cells typically regulate the embryonic development of DRGN and continue to exert a trophic influence into adulthood, though the underlying mechanism remains to be elucidated [32]. The intimacy of satellite cell/DRGN interrelationships is refined by the reciprocal projection of micro-villi from each cell deep into the respective cytoplasm of the other [33]. Indeed, Schaeffer and colleagues (2010) [7] proposed, after a chronic SN constriction injury that selectively activates apoptosis of satellite cells, the existence of either a paracrine or a cross-talk mechanism in apoptotic satellite cells which activates pro-survival factors in associated DRGN. Therefore, we suggest that the loss of target-derived neurotrophic factors combined with depletion of satellite cell-derived local trophic support [34] are key determinants of DRGN apoptosis and probably contribute to the DRGN loss.

To further investigate the role of CASP2 in DRGN and DRG glia apoptosis, we have used a well-established *in vitro* DRG model that provides a mixed population of axotomised adult DRGN and DRG glia that undergo apoptosis when serum is withdrawn. This primary cell culture model mimics the loss of target-derived neurotrophic factors experienced by DRGN after a procedure that transects the SN and prevents re-innervation. Our *in vitro* serum-withdrawal model detected significant levels of C-CASP2 in TUNEL^+^ DRGN and DRG glia consistent with our *in vivo* observations, linking DRGN and DRG glia death with CASP2. Although earlier reports suggest that CASP2 is processed after a variety of stimuli but was not required for death of sensory DRGN [35], these studies were performed in postnatal 2-day old rats, which are NGF-dependent for their survival. This is contrast to our current study where we use adult DRGN, which are independent of NGF for their survival and generally less reliant on trophic support [27] and show that DRGN death is regulated by CASP2 in both *in vivo* and *in vitro* serum withdrawal paradigms. Furthermore, we found little or no C-CASP3 immunoreactivity in serum-starved DRGN, while an abundance of C-CASP2 immunoreactivity was observed in these neurons, further vindicating our assertion that CASP2 is the major caspase responsible for death of DRGN after neurotrophic factor withdrawal.

The sub-cellular localization of C-CASP2 and the mechanism of activation of CASP2 may be linked and differs from cell type and death stimulus. Although the majority of CASP2 is found in the cytoplasm [36] some contradictory findings report that pro-CASP2 is localized to both cytoplasm and nucleus and that processing occurs in both compartments. CASP2 is constitutively localised to the nucleus by interaction of its nuclear localization signal with both its prodomain signal and the importin mediator [36], [37]. Our results are consistent with these observations since they demonstrate that, although C-CASP2 is both nuclear and cytoplasmic in DRGN, significantly greater levels were detected in the nucleus after serum withdrawal. Colussi and co-workers (1998) [36] also reported that CASP2 was localised in the cytoplasm, mitochondria and nuclei of normal Jurkat cells, while C-CASP2 was only present in the nucleus of apoptotic cells. Thus the nucleus appears to be the site of CASP2 cleavage and activation, both of which remain nuclear even after the induction of cell death [38].

Of potential translational importance, we have also demonstrated that silencing of CASP2 expression by siRNA-mediated knockdown of CASP2 mRNA protected DRGN from apoptosis after serum withdrawal. By contrast, CASP3 knockdown failed to rescue DRGN from apoptosis. Therefore, we suggest that CASP2 plays a major role in the execution of apoptosis in axotomised adult DRGN after neurotrophic factor depletion. Despite CASP2 sharing features of both initiator and effector caspases, processed rapidly in response to both intra- and extra-cellular signalling events occurring early during apoptosis in many cell types [39], [40], [41], its role in apoptosis has been contentious. For example, O'Reilly et al. (2002) [35] reported that DRGN in postnatal 2 day-old CASP-2 deficient mice did not die after NGF withdrawal. However, this contradicts another study, which reported that decreasing CASP-2 levels by anti-sense technology, delayed apoptosis induced by trophic factor deprivation in sympathetic neurons [42]. Others have also shown that CASP2 triggers apoptosis of serum-deprived PC12 cells and cortical neurons, while CASP2 inhibitors and siRNA-mediated knock down of CASP2 protects these neurons from death induced by serum deprivation [43], [44]. Our current results however, agree with the vast body of evidence that suggest that CASP2 regulates the death of DRGN.

In conclusion, we have demonstrated an interdependence of DRGN and DRG survival and a link between the activation of CASP2 and apoptosis *in vivo and in vitro*. Taken together, our data support the notion that CASP2, not CASP3, plays both a direct and indirect role in DRGN death. These studies provide valuable insight into the fundamental mechanisms underlying axotomy-induced apoptosis of DRGN and implicate a role for both target- and satellite cell-derived neurotrophic factors in their normal physiology. They further suggest the therapeutic utility of CASP2 silencing after PN axotomy.
